# A Case of Neonatal Junctional Epidermolysis Bullosa With a LAMB3 Mutation: Diagnostic Journey and Interdisciplinary Management

**DOI:** 10.1155/crpe/7881859

**Published:** 2026-02-19

**Authors:** Prakash I. Tete, Aswathy D. S., Mohamed Rafi, Sushma B. J.

**Affiliations:** ^1^ Department of Neonatology, Latifa Women and Children Hospital, Dubai, UAE; ^2^ Department of Biochemistry, National Institute of Medical Sciences and Research, Nims University Rajasthan, Jaipur, India

**Keywords:** consanguinity, epidermolysis bullosa, junctional epidermolysis bullosa, LAMB3 mutation, neonatal blistering disorder

## Abstract

A 20‐year‐old primigravida mother delivered a male infant at 35 weeks of gestation via normal vaginal delivery. The infant, born to consanguineous parents (second‐degree cousins), had good Apgar scores (9 and 10 at 5 and 10 min) and was appropriate for gestational age with stable postnatal vitals. Family history was notable for the maternal uncle’s death in 2013 due to epidermolysis bullosa (EB) complicated by sepsis. On Day 2 of life, the neonate developed peeling of the skin over the fingers, buttocks, and thighs. Dermatological evaluation raised suspicion of EB, and conservative wound care was initiated. The infant developed recurrent blisters and required intermittent respiratory support and antibiotics. An interdisciplinary team including neonatologists, dermatologists, geneticists, and pediatric surgeons coordinated management. Electron microscopy of the skin biopsy revealed a plane of cleavage at the level of the lamina lucida, suggesting junctional epidermolysis bullosa (JEB) or EB simplex. Subsequent exome sequencing identified a pathogenic mutation in the LAMB3 gene, confirming a diagnosis of JEB. This case highlights the importance of early recognition, family history, and genetic testing in the diagnosis and interdisciplinary management of inherited blistering disorders in neonates.

## 1. Introduction

Epidermolysis bullosa (EB) is a group of genetically inherited skin disorders characterized by skin and mucosal fragility, leading to blister formation either spontaneously or following minor mechanical trauma. The underlying cause is mutations in genes encoding structural proteins critical for skin integrity [1]. These blisters can affect not only the skin but also the mucosal linings of the respiratory and gastrointestinal tracts, eyes, and genitourinary system.

EB is classified based on the ultrastructural level of skin cleavage:

• EB simplex (intraepidermal)
• Junctional EB (intralamina lucida)
• Dystrophic EB (sublamina densa)
• Kindler syndrome (mixed levels)

Junctional epidermolysis bullosa (JEB) is a severe form caused by mutations in genes such as LAMB3, LAMA3, LAMC2, COL17A1, ITGA6, and ITGB4, which affect the lamina lucida of the epidermal basement membrane [2]. The incidence of JEB is rare, estimated at 3.6 to 6.7 per million per year [3, 4]. It typically presents at birth or shortly after, is autosomal recessive, and affects all sexes, races, and ethnic groups equally [5, 6]. The diagnosis is confirmed through skin biopsy using immunofluorescence antigen mapping or electron microscopy to identify the cleavage level, with definitive classification achieved through DNA‐based mutational analysis [7, 8]. A prognosis varies significantly by subtype, ranging from lethal in infancy in severe forms (JEB‐Herlitz) to survival into adulthood with moderate disability in intermediate forms.

## 2. Case Report

A 20 year‐old primigravida mother gave birth to a male infant at 35 weeks of gestation. The parents were second‐degree cousins. Maternal serology was negative, and an antenatal scan performed at 22 weeks was normal. The family history was significant; a maternal uncle died in 2013 from complications of EB, specifically extensive skin infection and sepsis. The baby was delivered via normal vaginal delivery with Apgar scores of 9 and 10 at 5 and 10 min, respectively.

The baby was appropriate for his gestational age and had stable vitals. On Day 2 of life in the postnatal ward, he was found to have peeling of the skin on his fingers, buttocks, and thighs (Figures 1(a) and 1(b)). A dermatology consultation was obtained, and a clinical impression of EB was made. Conservative management, including meticulous wound dressing, was initiated, and a skin biopsy was sent for electron microscopic study. New blisters continued to appear on different sites of his body during his stay in the NICU. An interdisciplinary team of neonatologists, dermatologists, geneticists, and pediatric surgeons was involved in his management.

Figure 1(a, b) Peeling of skin in fingers.

**Figure:**
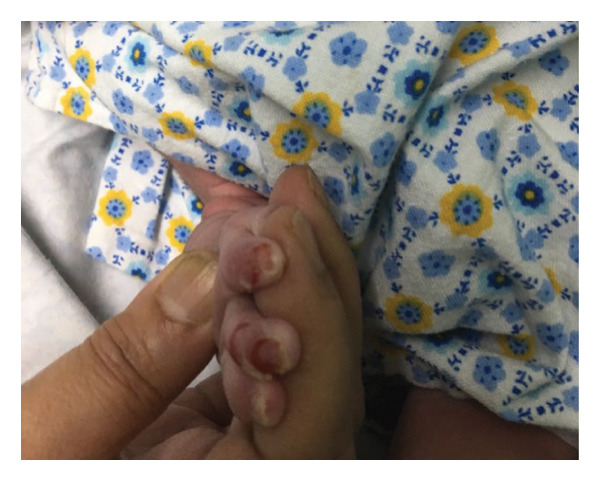
(a)

**Figure:**
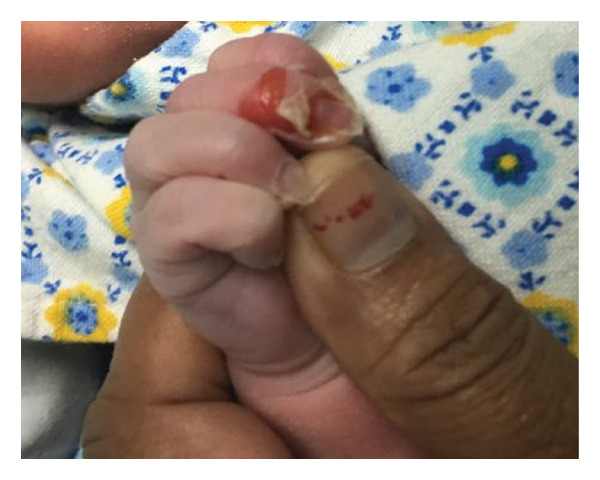
(b)

The infant required intermittent respiratory support and multiple courses of antibiotics based on clinical findings and laboratory reports. The electron microscopy report of the skin biopsy stated the following: “The specimen shows the lamina densa to be mainly attached to the dermal side of the epidermal separation, therefore implying a split at the level of the lamina lucida. The differential diagnosis for these changes includes JEB and its variants, as well as EB simplex.” Subsequent exome sequencing identified a pathogenic mutation in the LAMB3 gene, confirming the diagnosis of JEB.

### 2.1. Diagnosis and Subtype Classification in This Case

In this patient, the diagnosis of JEB was confirmed through a combination of clinical presentation, family history, and specialized testing. Electron microscopy was pivotal, demonstrating a cleavage plane at the lamina lucida. The definitive diagnosis was achieved through exome sequencing, which identified a pathogenic mutation in the LAMB3 gene. Based on the mutated gene (LAMB3) and the early, generalized presentation with significant skin involvement, this case is consistent with a severe form of JEB. The specific classification as Severe (Herlitz) or Intermediate JEB could not be definitively determined at this early neonatal stage, but the clinical course and genetic findings were most suggestive of the severe phenotype.

### 2.2. Inheritance and Counseling Applied to the Family

Given the autosomal recessive inheritance of LAMB3‐associated JEB and the consanguineous relationship of the parents, genetic counseling was provided. The parents were informed that they are both obligate carriers of the LAMB3 mutation and that each future pregnancy would carry a 25% risk of being affected by JEB. The option of prenatal diagnosis in future pregnancies through chorionic villus sampling or amniocentesis was discussed.

### 2.3. Management, Treatment, and Clinical Course

The infant’s management was coordinated by an interdisciplinary team. The cornerstone of his care was meticulous dermatological management, which involved gentle handling, protective padding, lancing and draining of new blisters with a sterile needle, and daily wound care with nonadherent dressings to prevent secondary infection.

Specific challenges encountered in his management included the following:

• *Fluid and Electrolyte Balance*: He experienced significant transcutaneous fluid and electrolyte loss through denuded skin, requiring careful monitoring and supplemental intravenous fluid support.
• *Nutrition*: To counter a hypercatabolic state and support healing, he was provided increased caloric intake via nasogastric tube feeds.
• *Pain Management*: He required scheduled acetaminophen and as‐needed morphine for procedural pain and background discomfort.
• *Infections*: He was treated with multiple courses of intravenous antibiotics for suspected secondary bacterial skin infections based on clinical signs.

Future treatment plans for this infant, assuming he survives the critical neonatal period, will focus on aggressive nutritional support, management of anemia, prevention of contractures, and monitoring for extracutaneous complications, particularly of the respiratory and gastrointestinal tracts. While gene‐targeted therapies were discussed with the family as a promising future avenue [9, 10], they were not a therapeutic option during his initial hospital stay.

### 2.4. Prognosis for This Patient

The prognosis for this infant remains guarded. The confirmation of a LAMB3 mutation, the early and generalized nature of his blistering, and the significant supportive care requirements align with a severe phenotype of JEB. The mortality rate in the first few years of life is high for severe JEB, often due to sepsis, failure to thrive, or respiratory complications. The interdisciplinary care he is receiving is aimed at maximizing his quality of life and managing complications, but there is currently no cure for his condition [11, 12].

## Author Contributions

Dr. Prakash I. Tete: conceptualization, clinical management, writing–original draft, and supervision.

Dr. Aswathy D. S.: data curation and writing–review and editing.

Dr. Mohamed Rafi: methodology, investigation, and clinical interpretation.

Dr. Sushma B. J.: biochemical analysis interpretation, writing–review and editing, and validation.

The manuscript was reviewed and verified entirely by the authors for accuracy and integrity.

## Funding

This study did not receive any specific grant from funding agencies in the public, commercial, or not‐for‐profit sectors.

## Disclosure

We confirm that this manuscript has not been published previously, is not under consideration elsewhere, and all authors agree with its submission to Case Reports in Pediatrics.

## Ethics Statement

This case report has been prepared in accordance with the CAse REport (CARE) Guidelines. The completed CARE checklist has been submitted along with this revised version.

## Consent

Written informed consent was obtained from the parents for publication of this case and the identifiable clinical image (Figure 1). A copy of the consent form is available with the corresponding author and can be provided to the journal on request.

## Conflicts of Interest

The authors declare no conflicts of interest.

## Data Availability

The data that support the findings of this study are available on request from the corresponding author. The data are not publicly available due to privacy or ethical restrictions.
